# Cellulose-Based Conductive Hydrogels: Design Strategies and Applications in Flexible Electronics

**DOI:** 10.3390/gels12050372

**Published:** 2026-04-29

**Authors:** Xu Dong, Mizhao Song, Zhihui Sui, Shuzhen Gao, Zhouyuanye Wan, Jianhua Zheng, Hongbin Li

**Affiliations:** 1 College of Light Industry and Textile, Qiqihar University, 42 Culture Street, Qiqihar 161006, China; 03020@qqhru.edu.cn (X.D.); jianhua0904@163.com (J.Z.); 2 Engineering Research Center for Hemp and Product in Cold Region of the Ministry of Education, Qiqihar 161006, China

**Keywords:** cellulose, conductive hydrogels, flexible functional materials, applications

## Abstract

With the rapid advancement of artificial intelligence and wearable technologies, the demand for soft, multifunctional electronic materials has grown substantially. Hydrogels have emerged as a promising platform due to their intrinsic softness, stretchability, and biocompatibility. Among them, cellulose-based conductive hydrogels uniquely integrate the sustainability of natural polymers with tunable electrical functionality, offering significant potential for flexible and biointegrated electronics. This review provides a comprehensive and critical perspective on the recent progress in cellulose-based conductive hydrogels. We systematically summarize key design strategies, including physical and chemical crosslinking and interpenetrating network engineering. More importantly, we present a comparative analysis of distinct conductive mechanisms, including ionic conduction, conductive polymers, metallic nanostructures, and carbon-based fillers, highlighting the inherent trade-offs among electrical conductivity, mechanical robustness, and environmental stability. Emerging applications in flexible electronics, energy storage, bioelectronics, and self-powered systems are discussed through structure–property relationships. Finally, we outline current challenges and future directions, emphasizing multifunctional integration, scalable fabrication, and long-term operational stability, thereby providing a framework for the rational design of next-generation sustainable electronic materials.

## 1. Introduction

Fossil resource depletion and escalating environmental pollution pose significant challenges to sustainable development in the context of global carbon neutrality goals [1,2,3]. This has accelerated the transition toward renewable and sustainable alternatives, with natural polymers emerging as promising candidates [4,5,6]. Such materials are critical to advancing green technologies and enabling a circular bioeconomy [7,8,9]. Cellulose, the most abundant renewable and biodegradable natural polymer, exhibits physicochemical properties that make it particularly valuable in this context [10,11]. Rich in hydroxyl and other reactive functional groups, cellulose enables extensive chemical modification and structural tuning [12,13]. Moreover, cellulose demonstrates excellent hygroscopicity, elasticity, and mechanical robustness, contributing to its superior processability compared with other biomass-based materials [14,15]. These inherent features have led to the widespread exploration of cellulose and its derivatives in fields such as biomedical engineering and flexible electronics [16,17,18]. Consequently, cellulose serves as a key material for environmentally sustainable and high-performance functional systems [19,20].

A hydrogel is a three-dimensional polymer network formed by crosslinking hydrophilic polymers in aqueous media, with water acting as the dispersion medium [21,22]. The unique structural characteristics of hydrogels endow them with exceptional physical and chemical properties [23,24,25]. Conductive hydrogels, as a class of functional materials, integrate hydrophilic polymer matrices with conductive components to achieve combined flexibility and conductivity [26,27,28,29]. These materials not only retain the high-water content and excellent biocompatibility typical of conventional hydrogels, but also exhibit superior electrical conductivity, making them highly promising for applications in supercapacitors, biosensors [30,31], and flexible wearable electronics [32,33]. Petroleum-based conductive hydrogels exhibit high flexibility, tunable mechanical properties, and excellent electrochemical performance, yet their limited biodegradability poses environmental concerns. This contradictory feature has driven researchers to explore more sustainable, eco-friendly alternatives.

Cellulose and its derivatives possess a high density of polar functional groups, including hydroxyl and carboxyl moieties. These groups facilitate ion transport and enhance the electrical conductivity and mechanical properties of hydrogels via coordination with metal ions [34,35]. Capitalizing on these distinctive properties, cellulose-based materials have emerged as promising candidates for the development of multifunctional conductive hydrogels [36]. Cellulose-based hydrogels are sustainable soft materials with intrinsic biodegradability, mechanical robustness, high water retention, recyclability, and biocompatibility, enabling broad applications [37,38]. Despite progress, studies remain largely descriptive and lack systematic comparison across material systems. Structure–property relationships governing conductivity, mechanical performance, and environmental stability remain poorly unified, hindering rational design.

**Figure 1:**
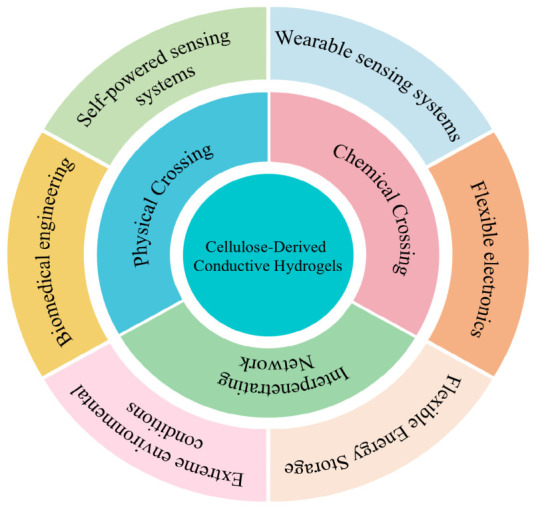
Schematic illustration of fabrication strategies and application domains of cellulose-based conductive hydrogels.

Here, we present a critical comparative framework for cellulose-based conductive hydrogels (Figure 1). We classify design and fabrication strategies, elucidate conduction mechanisms, and identify key trade-offs across representative systems. By linking material design with performance requirements, this work guides the development of high-performance, sustainable hydrogel electronics.

## 2. Design and Optimization of Cellulosic Hydrogels

### 2.1. Physical Crosslinking Strategies

**Figure 2:**
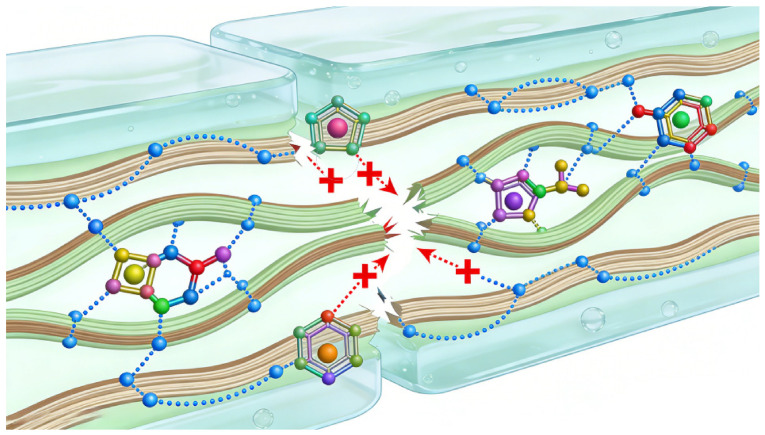
Reversible dissociation and recombination of non-covalent bonds in cellulose-based hydrogels.

Physical crosslinking enables the construction of cellulose-based hydrogels via reversible non-covalent interactions, including hydrogen bonding, electrostatic interactions, chain entanglement, metal–ligand coordination, and host–guest interactions. Unlike permanent covalent crosslinks, these dynamic interactions endow hydrogels with self-healing, shear-thinning, injectability, and adaptive mechanical compliance (Figure 2). The reversible dissociation and reformation of non-covalent bonds enable efficient energy dissipation under deformation while preserving network integrity, thereby reconciling softness with structural resilience.

Hydrogen bonding is a fundamental and versatile interaction in cellulose-based hydrogels, enabled by the high density of hydroxyl groups along the polysaccharide backbone [39]. The density, spatial distribution, and hierarchical organization of hydrogen bonds dictate mechanical performance and multifunctionality. For example, tannic acid–cellulose systems show enhanced toughness and thermal stability via reinforced hydrogen-bond networks [40]. Incorporating polyvinyl alcohol with TEMPO-oxidized cellulose nanofibers improves interfacial interactions and enables continuous ion-transport pathways [41]. CNFs function as hydrogen-bond-rich nano-crosslinkers capable of forming dense sacrificial networks that dissipate mechanical energy and enhance adhesion [42]. Such hierarchical hydrogen-bond architectures also support multifunctional integration, including transparent stretchable electronics and thermosensitive devices [43]. However, precise regulation of hydrogen-bond dynamics remains challenging, particularly under complex conditions. This limitation highlights the need for quantitative control and real-time characterization to fully exploit hydrogen-bonded networks.

Electrostatic interactions provide an additional mechanism for constructing stimuli-responsive and conductive networks. In CNF/polymer composites incorporating lithium ions, Zhuang et al. [44] demonstrated that ionic modulation simultaneously enhanced stretchability (≈1200%), conductivity, and strain sensitivity. The coupling between ion-transport pathways and π-conjugated frameworks establishes hybrid conduction networks capable of efficient signal transduction. However, maintaining electrostatic stability under fluctuating ionic environments remains a key challenge.

Host–guest interactions introduce molecular recognition capability and programmable reversibility into cellulose matrices. Jiang et al. [45] combined β-cyclodextrin-modified cellulose with adamantane derivatives and dynamic acyl hydrazone bonds to fabricate pH-responsive, self-healing supramolecular hydrogels. This approach highlights how molecular-scale inclusion complexes can precisely regulate network topology and responsiveness.

Beyond these classical interactions, emerging strategies such as multivalent ion exchange [46], Schiff-base coordination and Fe^3+^ complexation [47], and ion–water stabilization mechanisms for antifreezing tolerance [48] further expand the design space. Bioinspired architectures mimicking dermal structures have also been realized through synergistic physical interactions, achieving high fracture energy and antibacterial functionality [49].

Overall, physically crosslinked cellulose hydrogels rely on dynamic sacrificial networks and hierarchical non-covalent interactions to enable energy dissipation, self-healing, and environmental adaptability. Future progress requires quantitative control of interaction density, real-time characterization of bond dynamics, and multiscale modeling to predict structure–property relationships.

### 2.2. Chemical Crosslinking Strategies

**Figure 3:**
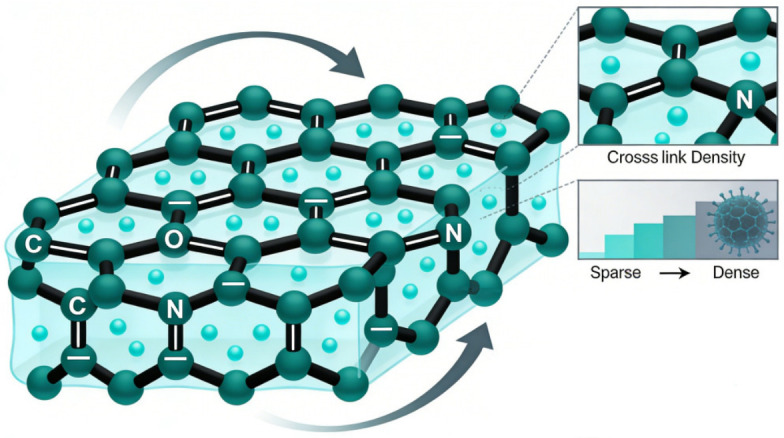
Cellulose-based hydrogels forming network structures through permanent covalent bonds.

Chemically crosslinked cellulose hydrogels feature permanent covalent crosslinks that confer superior mechanical strength, fatigue resistance, and environmental stability compared with physically crosslinked systems (Figure 3). Stable network architectures enable controlled swelling, long-term durability under cyclic deformation, and resistance to variations in temperature, pH, and ionic strength. Rational control of crosslinking chemistry, reaction kinetics, and cellulose functionalization enables precise tuning of network density and topology for targeted applications. For example, Farooq et al. [50] fabricated a zwitterionic cellulose nanofiber-reinforced poly(sulfobetaine methacrylate–acrylic acid–acrylamide) hydrogel via blue-light-initiated radical polymerization. The synergistic incorporation of zwitterionic moieties and cellulose nanofibers enabled simultaneous enhancement of mechanical strength and electrical conductivity, while imparting antibacterial activity against Escherichia coli and Staphylococcus aureus. This study illustrates how covalent network stabilization can coexist with functional conductivity and bioactivity. Structural reinforcement strategies further extend performance limits. Zhang et al. [51] generated self-reinforcing bacterial cellulose gels through epichlorohydrin-mediated covalent bridging, followed by cyclic stretching to induce nanofiber alignment. The combination of chemical crosslinking and post-processing-induced orientation significantly enhanced anisotropic mechanical properties, demonstrating the importance of coupling network fixation with structural organization. Dynamic covalent chemistry introduces reversibility into otherwise permanent networks. Yang et al. [52] designed dual-responsive hydrogels incorporating reversible acylhydrazone and disulfide bonds, achieving ≈96% self-healing efficiency while maintaining mechanical integrity under physiological conditions. The reversible network enabled pH- and redox-triggered sol–gel transitions and controlled drug release, exemplifying how dynamic covalent linkages bridge stability and adaptability.

Chemically crosslinked cellulose hydrogels are evolving from mechanically robust, static materials into multifunctional, stimuli-responsive systems. The integration of nanofiber reinforcement, network orientation engineering, and dynamic covalent chemistry enables concurrent optimization of strength, conductivity, antibacterial activity, and therapeutic delivery. Remaining challenges include minimizing cytotoxic crosslinkers, balancing permanent stability with dynamic reversibility, and developing green, scalable synthesis routes supported by predictive modeling frameworks.

### 2.3. Interpenetrating Network Structures

**Figure 4:**
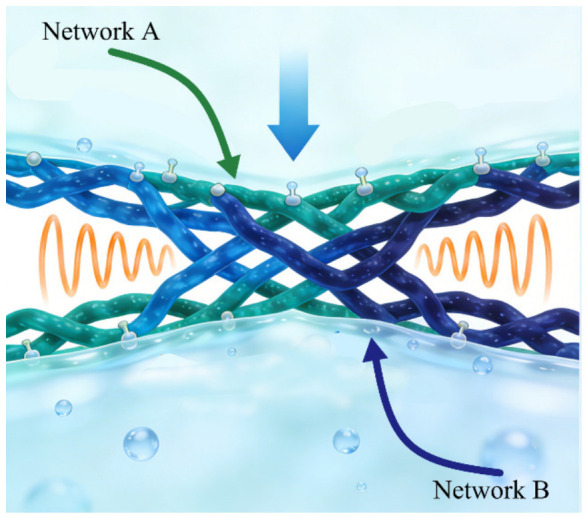
Interpenetrating polymer networks (IPNs) of cellulose-based hydrogels.

Interpenetrating polymer networks (IPNs) represent an advanced architectural paradigm for achieving synergistic reinforcement in cellulose-based hydrogels. By intertwining two or more polymer networks via topological entanglement and physical interlocking, IPNs preserve the intrinsic attributes of each component while enabling cooperative mechanical and functional enhancement. This architecture effectively decouples energy dissipation from elastic recovery, overcoming the limitations of single-network systems (Figure 4). Rahmani et al. [53] demonstrated that hydrophobic-association crosslinked polyacrylamide reinforced with cellulose nanocrystals and polyaniline achieved ultrahigh stretchability (≈2400%) with low modulus (≈30 kPa) and high conductivity (21.7 S m^−1^). The interpenetrating conductive network reconciled skin-like compliance with efficient charge transport, underscoring the mechanical–electrical synergy of IPN design. Hierarchical nanosheet assembly further enhances structural cohesion. Yan et al. [54] constructed polydopamine–graphene–cellulose hydrogels via exfoliation–assembly integration, forming physically and chemically interpenetrated networks with improved conductivity and mechanical robustness. Wei et al. [55] introduced a multi-modulus strategy combining chemically crosslinked cellulose domains with physically crosslinked graphene oxide nodes, enabling concurrent increases in strength and toughness alongside photothermal antibacterial functionality. A distinct dual-network paradigm reported by Ye et al. [56] combined sparse chemical crosslinks with dense reversible hydrogen bonds. In this configuration, sacrificial hydrogen bonds dissipate energy under stress, while the loosely crosslinked covalent framework ensures elastic recovery. Force-induced alignment produced optical anisotropy, translating mechanical deformation into optical signals. In conductive IPN systems, Wang et al. [57] integrated regenerated bacterial cellulose with polypyrrole and carbon nanotube networks to establish thermally stable, electrically conductive interpenetrating architectures suitable for flexible electronics. Beyond electronics, Dash et al. [58] demonstrated that gelatin–oxidized cellulose nanowhisker IPNs provide enhanced storage modulus and thermal stability for tissue engineering scaffolds.

Collectively, interpenetrating network (IPN) architectures enable multiscale synergy among rigidity, elasticity, conductivity, and environmental responsiveness. Tuning network composition, crosslink density, nanoscale reinforcement, and interpenetration degree enables performance regimes inaccessible to single networks. Future progress requires scalable fabrication, quantitative control of network interlocking, and multiscale modeling to guide rational IPN design for biomedical, electronic, and environmental applications.

## 3. Construction of Cellulose-Based Conductive Hydrogels

### 3.1. Ion Conduction Systems

**Figure 5:**
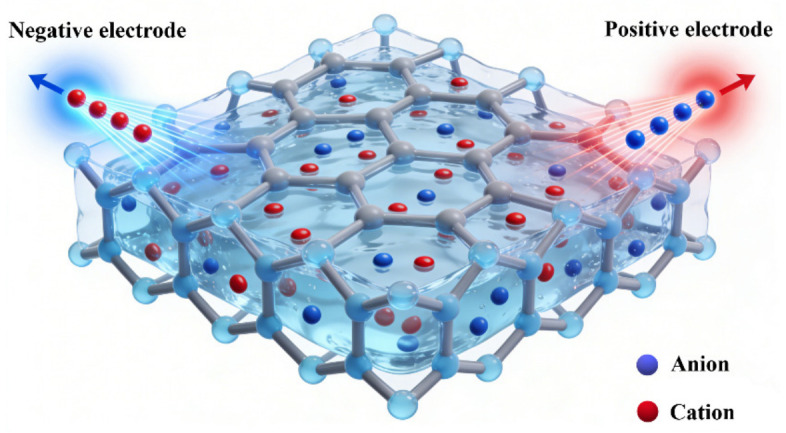
Schematic diagram of the conductive mechanism in ion-conductive cellulose-based hydrogels.

Ion-conductive cellulose hydrogels rely on hydrated three-dimensional polymer networks containing mobile ionic species that migrate under applied electric fields (Figure 5) [59]. Their electrical performance is governed by ionic concentration, mobility, and the continuity of ion-transport pathways, closely resembling charge conduction in biological tissues [60,61]. This biomimetic mechanism underpins their widespread exploration in bioelectronics, wearable sensing, and human–machine interfaces.

Recent advances reveal that high-performance ion conduction arises from synergistic regulation of ionic environments and hierarchical network architecture. Hydrophobic association strategies, for example, introduce phase-separated domains that reinforce the polymer backbone while maintaining ion mobility. Wahab et al. [62] demonstrated that combining micelle-induced physical crosslinking with lithium salt doping produced a hydrogel exhibiting both high ionic conductivity (320 S m^−1^) and rapid response (≈17 ms), illustrating how decoupling mechanical reinforcement from ion transport can mitigate the traditional softness–conductivity trade-off.

Multivalent ion coordination further enhances network stability and transport efficiency. Li et al. [63] employed Al^3+^-mediated coordination within a PVA/CNF organohydrogel matrix, where trivalent ions simultaneously reinforced the network and facilitated ion migration. The resulting system maintained stable sensing performance across extreme temperatures and prolonged environmental exposure, highlighting the importance of dynamic coordination chemistry in constructing stable ionic channels.

Beyond mechanical reinforcement, multifunctional integration has also been realized. Sha et al. [64] incorporated Zn^2+^/Al^3+^ ionic networks into porous PVA–CNF hydrogels and coupled them with triboelectric nanogenerators, enabling self-powered strain sensing. Similarly, Wu et al. [65] engineered multicomponent ionic environments incorporating dissolved cellulose and bimetallic ions, achieving simultaneous enhancement of compressive strength and ionic conductivity. Collectively, these studies suggest that ionic transport efficiency is maximized when ion–polymer coordination, solvent retention, and structural hierarchy are co-optimized.

Nevertheless, a fundamental challenge remains in balancing ionic concentration with physiological compatibility. High salt contents improve conductivity but may induce osmotic imbalance, ion diffusion, and long-term instability in biointerfaces. Future directions should therefore focus on immobilized ionic reservoirs, partially coordinated ion clusters, and bioinspired ion-channel architectures that achieve high conductivity without compromising biosafety.

### 3.2. Composite Conductive Packings

#### 3.2.1. Conducting Polymers

Conducting polymers, defined by delocalized π-electron backbones, transform insulating polymer matrices into electronically active systems with tunable conductivity [66]. Representative materials—including polyaniline [67], poly(3,4-ethylenedioxythiophene) [68], and polypyrrole [69,70]—enable percolative electronic networks whose transport characteristics depend on doping level and microstructural ordering.

When integrated into cellulose hydrogels, conducting polymers establish electronically continuous pathways while preserving hydration and mechanical compliance. Importantly, nanoscale morphology control has emerged as a central design principle. Han et al. [71] demonstrated in situ nanofiber formation within hydrogel matrices, mitigating charge-transport limitations without sacrificing flexibility. Likewise, anisotropic alignment strategies, as reported by Jiang et al. [72], show that directing conductive polymer growth along aligned cellulose fibrils enables directional charge transport and improved biointerface compatibility. Device-level integration further highlights the multiscale nature of conductive optimization. Li et al. [73] constructed textile-based supercapacitors integrating polyaniline-coated electrodes with cellulose-reinforced electrolytes, where stress-matched interfaces improved durability and electrochemical stability. These examples collectively indicate that conductive-polymer–hydrogel hybrids achieve non-additive performance gains through coordinated optimization across molecular (π–π stacking), mesoscale (interpenetrating networks), and macroscopic (device integration) levels.

#### 3.2.2. Metal Nanomaterials

Metallic nanostructures introduce continuous electron-transport pathways and high carrier mobility into hydrogel systems. Nanoparticles [74], nanowires [75], and nanorods [76,77] act as conductive scaffolds to enhance electrical and catalytic performance.

Mechanistically, performance enhancement arises from interfacial charge transfer and dynamic ion–metal interactions. Lee et al. [78] embedded liquid metal microparticles within cellulose hydrogels, where controlled gallium ion release generated additional ionic crosslinking, while metallic domains provided continuous electron transport. The dual ionic–electronic conduction mechanism strengthened both mechanical and electrochemical performance. Hierarchical surface engineering further amplifies sensitivity. Yue et al. [79] constructed carbonized ZIF-8-derived protrusive architectures that formed dense contact–separation interfaces, achieving ultrahigh strain sensitivity (GF = 15,901) and rapid response. These findings illustrate how nanoarchitectural design directly governs charge-transfer efficiency and sensing amplification. Despite their advantages, metallic nanomaterials raise concerns regarding ion leaching, oxidative degradation, and long-term cytocompatibility. Surface functionalization, encapsulation, and biodegradable metallic systems are key to translating metallic-hybrid hydrogels into clinical and implantable applications.

#### 3.2.3. Carbon Materials

Carbon nanomaterials—including graphene derivatives [80], carbon nanotubes [81], and MXene [82,83,84]—provide high electrical conductivity, mechanical robustness, and tunable surface chemistry, enabling multifunctional reinforcement within cellulose matrices. A recurring design principle is interfacial stabilization combined with hierarchical dispersion control. Yu et al. [85] stabilized MXene within PVA matrices via polydopamine-mediated protection, suppressing oxidation while maintaining high conductivity. Yang et al. [86] utilized tannic-acid-modified nanofillers to introduce hydrogen bonding, π–π stacking, and coordination interactions, thereby enhancing toughness and strain sensitivity. Sustainable dispersion strategies further improve cytocompatibility and processability. Usala et al. [87] developed a colloidal rGO–CNF bioink suitable for extrusion-based bioprinting, while Li et al. [88] developed a multifunctional poly(vinyl alcohol)–borate/boric acid–graphene oxide–cellulose nanofiber (PBGC) conductive hydrogel, in which the incorporation of GO and CNF significantly enhances electrical conductivity and strain sensitivity.

Collectively, carbon-reinforced hydrogels demonstrate that precise nano–bio interface engineering is essential for achieving high conductivity without sacrificing mechanical resilience or biological compatibility. Remaining challenges lie in scalable manufacturing, interface reproducibility, and long-term stability.

### 3.3. Interpenetrating Conductive Polymer Networks

IPN strategies represent a unifying framework for integrating ionic and electronic conduction within a single hydrogel matrix. By decoupling mechanical reinforcement from conductive pathways, IPNs overcome conventional trade-offs between strength and electrical performance.

Recent designs employ synergistic combinations of hydrophobic association, dynamic coordination bonds, and multivalent ions to construct hierarchical interaction networks [89,90,91]. A representative example is the PEDOT:PSS-based IPN system reported by Zhao et al. [92], in which π–π stacking, electrostatic interactions, and polymer entanglement collectively establish efficient electron-transport pathways while dissipating mechanical energy. The resulting hydrogels exhibit high elongation (1060%), tensile strength (463 kPa), and stable multimodal sensing capabilities, including ECG and EMG monitoring. More broadly, IPN architectures highlight a central principle in conductive hydrogel design: multiscale interaction engineering enables simultaneous optimization of conductivity, mechanical durability, and biointerface stability. As wireless integration and intelligent sensing technologies continue to evolve, IPN-based cellulose hydrogels are poised to play a pivotal role in next-generation human–machine interfaces and bioelectronic systems.

Despite advances, balancing electrical, mechanical, and environmental performance remains a central challenge in conductive hydrogels. Ionic systems offer flexibility and biocompatibility but are limited by dehydration and ion diffusion. Conductive polymers enhance electronic transport but suffer from brittleness and doping instability. Metallic nanomaterials provide high conductivity but raise concerns over oxidation, ion leakage, and cytocompatibility. Carbon-based systems offer balanced performance but are constrained by scalability and dispersion; no single strategy suffices, and progress requires integrated multicomponent systems to reconcile these trade-offs.

## 4. Applications in Flexible Electronics

### 4.1. Energy Storage Electronics

**Figure 6:**
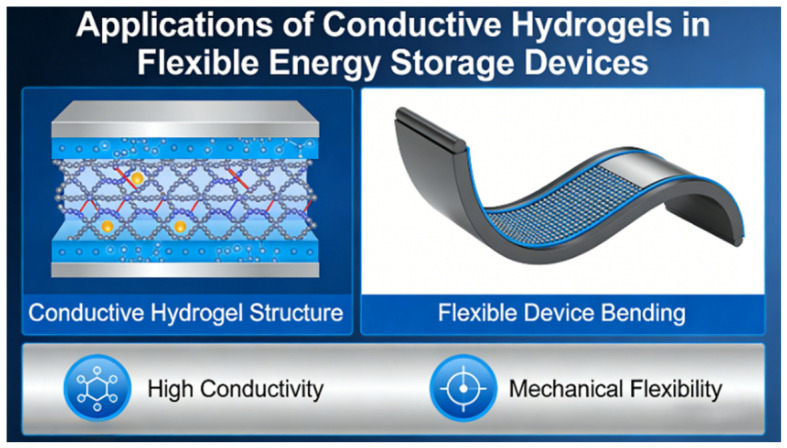
Application of cellulose-based conductive hydrogels in flexible energy storage devices.

The rapid evolution of flexible and wearable electronics imposes stringent requirements on electrochemical systems to maintain stable energy output under repeated bending, stretching, and shear. Cellulose-based conductive hydrogels have emerged as promising platforms for electrolytes and electrodes, owing to their ability to couple mechanical compliance with continuous ion and electron transport (Figure 6).

In aqueous zinc-ion systems, dendritic growth and parasitic side reactions remain critical barriers to long-term stability. Zhang et al. [93] addressed this challenge by engineering an amphoteric cellulose-based dual-network hydrogel electrolyte featuring zwitterionic ion-transport channels. The spatial homogenization of Zn^2+^ flux promoted preferential deposition along the (002) crystal plane, while carboxyl-functional groups facilitated desolvation of [Zn(H_2_O)_6_]^2+^ complexes. This dual-regulation strategy exemplifies how molecular-level channel engineering can suppress dendrite formation and interfacial degradation, establishing a blueprint for stable aqueous metal batteries. Beyond dendrite regulation, multifunctional energy devices increasingly integrate structural resilience with electrochemical performance. Wang et al. [94] developed a sandwich-structured hydrogel supercapacitor incorporating cellulose, phytic acid, and polyaniline, achieving an areal energy density of 168.2 μWh cm^−2^ and retaining 89% capacitance after 1000 bending cycles. Hierarchical interface design enables stress redistribution while preserving ion transport pathways, highlighting how mechanical–electrochemical decoupling improves durability. Thermoelectric conversion further expands the energy-harvesting landscape. Liu et al. [95] constructed bacterial cellulose ion gels integrating the ionic liquid [EMIm][DCA] through strong ion–polymer interactions. The resulting composite exhibited high ionic conductivity (2.88 × 10^−2^ S cm^−1^), an ionic thermovoltage of 18.04 mV K^−1^, and suppressed thermal conductivity (0.21 W m^−1^ K^−1^), enabling efficient ion thermoelectric conversion. Such systems highlight the emerging paradigm of hybrid ionic–thermal energy devices capable of self-powered operation.

Collectively, cellulose-based hydrogel energy systems demonstrate that interfacial ion regulation, hierarchical mechanical reinforcement, and multifunctional coupling are central to achieving high safety, adaptability, and environmental compatibility. Ongoing advances in smart electrolyte design and in situ composite assembly are steering flexible energy storage toward integrated, adaptive, and sustainable architectures.

### 4.2. Wearable Sensing Electronics

**Figure 7:**
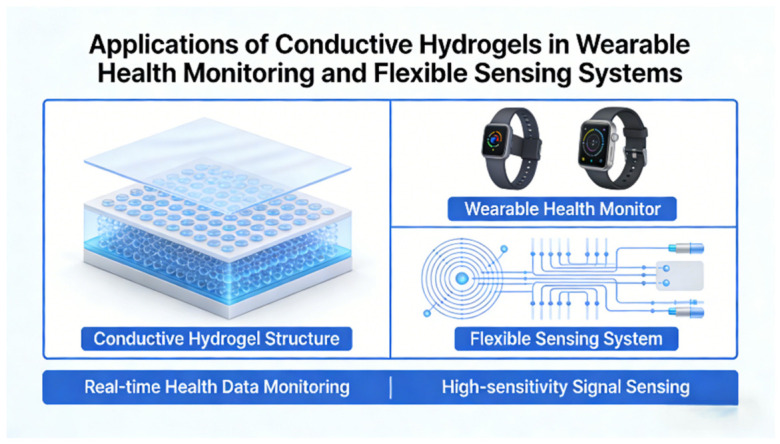
Application of conductive hydrogels in wearable health monitoring and flexible sensing systems.

The integration of mechanical flexibility, ionic conductivity, and environmental friendliness renders cellulose-based conductive hydrogels well suited for wearable health monitoring and flexible sensing systems (Figure 7). In flexible batteries, nanocellulose-reinforced hydrogel electrolytes provide shear-resistant frameworks that maintain ion-transport integrity under mechanical deformation. Wang et al. [96] demonstrated that a nanocellulose/polyacrylamide electrolyte enabled Zn–MnO_2_ cells to retain 88.3% capacity after 1000 cycles at 4 C, while sustaining stable performance under repeated sewing-induced shear stress. This study illustrates how structural skeleton–matrix synergy enhances mechanical tolerance without compromising electrochemical kinetics. Multimodal sensing further leverages hierarchical composite design. Zhao et al. [97] constructed an electronic skin by integrating hydroxypropyl cellulose, PACA, and carbon nanotubes, forming a multi-responsive hydrogel capable of detecting temperature, pressure, and strain while exhibiting chromatic variation induced by structural reconfiguration. Such systems exemplify how nanoengineered conductive pathways and dynamic bonding networks can translate microstructural deformation into amplified electrical and optical signals.

These developments collectively redefine flexible electronics as mechanically adaptive, self-healing, and environmentally responsive systems. Future research increasingly targets neural-interface-compatible hydrogels with hybrid ionic–electronic conduction, and self-powered smart bandages integrating sensing with localized therapeutic delivery.

### 4.3. Biointegrated Electronics for Tissue Engineering

**Figure 8:**
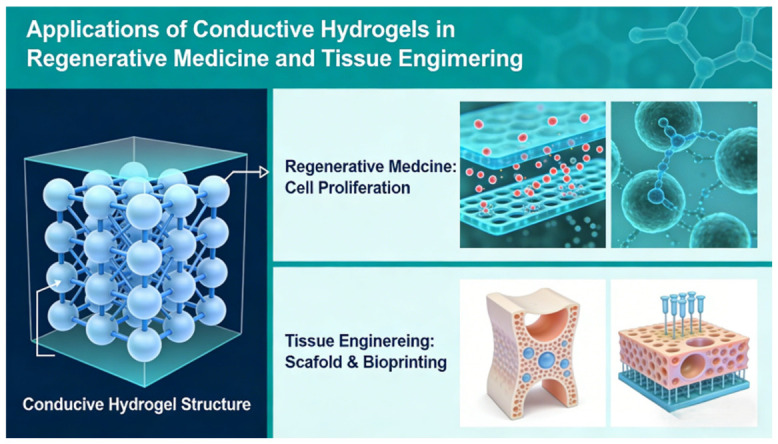
Applications of conductive hydrogels in regenerative medicine and tissue engineering.

Conductive hydrogels are driving a paradigm shift in regenerative medicine from passive scaffolds to active bioelectronic modulation. By mimicking the electrophysiological microenvironment of native tissues, these materials enable synchronized electrical signaling, enhanced cell migration, and controlled biochemical release (Figure 8).

In neural regeneration, Han et al. [98] fabricated digitally printed conductive scaffolds based on gelatin methacryloyl and chitosan-incorporating PEDOT nanoparticles. The conductive matrix promoted PC12 and Schwann cell proliferation, while electrical stimulation enhanced axonal elongation and functional recovery in vivo, demonstrating the therapeutic value of engineered electrical cues. Similarly, Amirabdollahian et al. [99] developed a silk fibroin/decellularized extracellular matrix hydrogel reinforced with carbon nanotubes, achieving enhanced conductivity (2.35 × 10^−4^ S m^−1^) and structural stability suitable for brain tissue engineering. Spencer et al. [100] further advanced bioprinted GelMA/PEDOT:PSS constructs, validating in vivo biocompatibility and controlled degradation profiles. Electrically triggered drug delivery adds another dimension of functional control. Ho et al. [101] incorporated reduced graphene oxide within PEGDA hydrogels, enabling voltage-dependent nanoparticle release (2–10 V) via electro-induced pore expansion. Meanwhile, Ouyang et al. [102] designed an injectable supramolecular nanoconductive hydrogel integrating growth factors, antimicrobial peptides, and single-walled carbon nanotubes, simultaneously promoting vascular remodeling, immune modulation, and endogenous electric-field-guided cell recruitment. Aligned conductive architectures further enhance regeneration. Yao et al. [103] fabricated oriented CNT/GelMA fibers replicating axonal anisotropy, where combined electrical stimulation significantly restored motor function in spinal cord injury models.

Taken together, these studies delineate three synergistic mechanisms that underpin tissue regeneration: enhanced intercellular electrical coupling, dynamic mechanical adaptability that facilitates structural remodeling, and electronically programmable spatiotemporal drug delivery. Future efforts should target neurotransmitter-responsive hydrogels, optogenetic integration, and organ-on-chip platforms to gain mechanistic insights, positioning conductive hydrogels as next-generation bioelectronic therapeutics.

### 4.4. Self-Powered Flexible Electronic Systems

The integration of conductive hydrogels with energy harvesting components establishes autonomous sensing systems that emulate biological energy self-sufficiency. Wang et al. [104] engineered a cationic nanocellulose/graphitic carbon nitride composite hydrogel featuring dynamic ion-interaction sacrificial bonds, enabling a microcapacitor to reach 15 V via simple mechanical tapping. Yang et al. [105] realized continuously 3D-printed conductive hydrogels with periodic minimal surface architectures capable of self-powered sensing under mechanical deformation.

Ion thermoelectric systems further exploit ambient thermal gradients. Zhang et al. [106] developed a calcium-ion crosslinking gradient hydrogel achieving an ionic thermovoltage of 34.27 mV K^−1^ and power density of 730 mW m^−2^, demonstrating efficient conversion of low-grade heat into electrical output. Jia et al. [107] enhanced proton conduction through ordered covalent organic framework–cellulose composites, maintaining conductivity at −40 °C and enabling high-performance gel supercapacitors and triboelectric nanogenerators.

Collectively, these innovations establish a design paradigm centered on environmental energy harvesting, hierarchical proton/ion transport regulation, and multifunctional device integration. Emerging research directions include biodegradable autonomous electronics, living hydrogels synthesized via microbial pathways, and data-driven inverse materials design.

### 4.5. Electronics for Extreme Environments

Conductive hydrogels tailored for extreme conditions are transforming specialized electronics from passive endurance to active adaptability [68]. Zhu et al. [108] introduced an ion-regulated cellulose hydrogel featuring electrostatic “ion-lock” interactions between nanocellulose carboxyl groups and metal ions, maintaining conductivity at −30 °C. Quan et al. [109] achieved operation at −80 °C via a double-crosslinked network incorporating LiCl and MXene, where Hofmeister effects and ion–polymer interactions suppressed freezing and dehydration. Han et al. [110] constructed glycerol/water dual-network hydrogels with antifreezing capability and developed a triboelectric nanogenerator integrated into intelligent insoles, achieving 97.1% classification accuracy of skiing maneuvers via machine learning. For arid environments, Lu et al. [111] designed a thermostable ion-gel sustaining conductivity under 7% relative humidity and functioning as a rapid-response Joule heater. Collectively, these studies highlight ion confinement, solvent engineering, and hierarchical porosity as key determinants of environmental resilience. Future efforts are expected to leverage AI-driven inverse design, modular architectures, and materials genome strategies to realize hydrogel electronics capable of autonomous adaptation under extreme conditions.

Despite these advances, device-level progress has yet to translate into practical deployment. The lack of standardized metrics for mechanical durability, electrical stability, and long-term biocompatibility remains a central bottleneck. Recent progress in manufacturing, conductive network engineering, and multiscale structure–property correlations provide a unified framework for flexible electronic architectures (Table 1), yet systematic validation under real-world conditions is still needed.

## 5. Conclusions, Perspectives, and Challenges

This Review provides a critical synthesis of cellulose-based conductive hydrogels, encompassing design strategies, conduction mechanisms, and applications. Performance is governed by intrinsic trade-offs among conductivity, mechanical robustness, and environmental stability, limiting simultaneous optimization. Physically and chemically crosslinked systems exhibit complementary strengths and limitations, while interpenetrating networks partially mitigate these constraints but remain challenged by synthetic complexity and scalability. These hydrogels show promise in wearable sensing, flexible energy storage, tissue engineering, and flexible electronics under extreme conditions. Practical deployment remains constrained by limited long-term stability, environmental resilience, and scalable fabrication. Key challenges remain the design of hierarchical conductive pathways and cost-effective fabrication. Future progress depends on integrated strategies combining molecular design, quantitative structure–property control, and scalable fabrication, along with deeper mechanistic understanding to enable translation into practical technologies.

## Figures and Tables

**Table 1:** Summary of representative cellulose-based conductive hydrogels: materials, conductivity, mechanical properties, and applications.

System Type	Key Materials	Conductivity (S m^−1^)	Mechanical Properties	Applications	Ref
Ion conduction	CNF/Al^3+^/PVA	High electrical conductivity	Stretchability (696.0%), strength (0.9 MPa)	Wearable sensors	[63]
Metal nanomaterial	Cellulose/liquid metal/metal nanoparticles/nanowires	49.63	Stretchability (334.0%), strength (3.0 MPa)	Ultrahigh sensitivity	[79]
Carbon material	Cellulose/MXene-	High conductivity	Mechanical reinforcement, toughness, flexibility	Electronic skins, energy storage	[112]
Interpenetrating polymer networks	Carboxylated cellulose nanofiber/chitosan/Ca^2+^	0.11	Stretchability (406.7%), strength (0.3 MPa)	Flexible electronics	[90]
Conducting polymer	Hydroxyethyl cellulose/PEDOT:PSS/PAM	1.44	Stretchability (1060%), strength (0.5 MPa)	flexible electronic signal detection, sensing system	[92]
Flexible energy storage	Bacterial cellulose/ionic liquid	2.88	Stretchability (50.0%), strength (3.1 MPa)	Thermoelectric devices	[95]
Tissue engineering	Cellulose nanofibrils/rGO	Positive response to electrical stimulation stimuli	Enhancement of physical stability	Complex tissues, particularly neural and cardiac tissues	[87]
Wearable health monitoring	Carboxymethylcellulose/ATAC/AMPS	8.20	Stretchability (>1701.0%), strength (3.1 MPa)	Flexible sensors	[113]
Extreme-environment electronics	Cellulose/PAM/Zn^2+^	8.89	Strength (0.31 MPa)	Flexible supercapacitors	[114]

## Data Availability

Not applicable.
